# Impact of the updating of clinical guidelines for RSV bronchiolitis on the use of diagnostic testing and medications in tertiary hospitals in Colombia

**DOI:** 10.11604/pamj.2022.42.219.24876

**Published:** 2022-07-20

**Authors:** Jefferson Antonio Buendía, Diana Guerrero Patiño

**Affiliations:** 1 Grupo de Investigación en Farmacología y Toxicología, Centro de Información y Estudio de Medicamentos y Tóxicos (CIEMTO), Departamento de Farmacología y Toxicología, Facultad de Medicina, Universidad de Antioquia, Medellín, Colombia,; 2 Hospital Infantil Concejo de Medellin, Medellin, Colombia

**Keywords:** Bronchiolitis, respiratory syncytial virus, salbutamol, nebulized hypertonic saline solution

## Abstract

**Introduction:**

the incidence of Respiratory Syncytial Virus (RSV) infection and their variability in the clinical management, make this disease a candidate for monitoring adequate use of resources. The objective of this study was to evaluate the impact of the updating of clinical guidelines for RSV bronchiolitis on the use of diagnostic testing and medications in tertiary hospitals in Colombia.

**Methods:**

we performed a cross-sectional study, evaluating the frequencies of drug prescription and medical tests, before (January-December 2016) and after (January to December 2019) of updating and dissemination of a new protocol for the treatment of RSV bronchiolitis in two tertiary hospitals in Colombia.

**Results:**

a total of 108 patients with RSV bronchiolitis were included. The demographic characteristics and clinical manifestations were similar in both groups. The length of hospital stays was similar in both groups. We did not find statistically significant differences in the frequency of medical tests. There was a decrease in the use of salbutamol (67.3% pre-protocol vs 51.8% post-protocol; P < .01). There were also significant reductions in the use of nebulized hypertonic saline solution (91.6% vs 82.6% P = 0.004).

**Conclusion:**

our results demonstrate that the updating of clinical guidelines for RSV bronchiolitis was effective, as it achieved decreases in the use of bronchodilators and nebulized hypertonic saline solution. It is necessary to continue developing new strategies targeted to increase adherence to guidelines and evaluate the impact on the use of resources.

## Introduction

Bronchiolitis causes significant morbidity and mortality in infants and young children worldwide [1,2]. Respiratory Syncytial Virus (RSV) is the most common cause of serious bronchiolitis and lower respiratory tract disease in infants and young [3]. The high medical costs associated with RSV infection impose a relevant economic burden, especially in tropical middle-income countries [4-6]. The frequencies of inappropriate use of health resources range up to 60% of patients [7]. In Colombia, a middle-income country in Latin America, a quarter of every dollar spent on this disease was spent on practices with a low level of evidence [8].

A possible explanation for this problem is the variability in recommendations for diagnosis and pharmacological management in bronchiolitis. Kirolos *et al*. in a systematic review of thirty-two clinical practice guidelines, found conflicting recommendations across many guidelines as to whether inhaled / nebulized bronchodilators should be trialed. Also, there were mixed recommendations for the use of nebulized epinephrine and corticosteroids [9]. Personal history or diagnosis of atopic dermatitis (OR 5.30; CI 95% 1.14-24.79), length of hospital stay (OR 1.48; CI 95% 1.08-2.03), and the number of siblings (OR 1.92; CI 95% 1.13-3.26) have been identified as independent predictors of inappropriate use of diagnostic tests and treatment of bronchiolitis [7].

In 2014, the ministry of health in Colombia following an explicit and evidence-based methodology publish the clinical practice guideline for bronchiolitis to be adopted by all hospitals in Colombia [10]; which adopt most of the recommendations of the 2006 American Academy of Pediatrics (AAP) bronchiolitis guidelines [11]. After 2014, new guides have been published that recommend limited use of diagnostic testing and medications, including bronchodilators, corticosteroids, and antibiotics [2]; and many hospitals update the guide according to this new evidence to contain the inappropriate use of resources [12]. The objective of this study was to evaluate the impact of the updating of clinical guidelines for RSV bronchiolitis in the use of diagnostic testing and medications in tertiary hospitals in Colombia.

## Methods

**Study design and setting:** we performed a cross-sectional study, evaluating the frequencies of drug prescription and medical tests, before (January-December of 2016) and after (January to December of 2019) an updating of clinical guidelines for RSV bronchiolitis in two tertiary hospitals in Colombia.

**Study participants:** inclusion criteria were defined as children younger than two years of age admitted to the pediatric ward with a diagnosis of bronchiolitis (ICD-10 code: J21.0) due to RSV confirmed using direct immunofluorescence (Light Diagnostics TM Respiratory Panel 1 DFA, Merck-Millipore Laboratory) [13]. Patients without lower respiratory compromise, with positive bacterial cultures on admission, and confirmed whooping cough (culture or PCR) were excluded.

**Procedures:** medical records of all patients admitted with bronchiolitis to the emergency department were reviewed. We collected the following variables: age, sex, weight, height, signs, and symptoms on admission (including fever, chest indrawing, chest auscultation, %SPO_2_), vaccination scheduled chart for age, current exposure (maternal or paternal) to cigarette smoking, history of prematurity and bronchopulmonary dysplasia confirmed by a neonatologist at the time of discharge from the NICU, comorbidities (congenital heart disease, neurological disease), diagnostic tools as chest X rays, hemograms, etc. Additionally, we collected variables related to outcomes of care or disease-severity parameters such as length of hospital stay. RSV was confirmed using direct immunofluorescence (Light Diagnostics TM Respiratory Panel 1 DFA, Merck-Millipore Laboratory). NPA data for other viruses were not available at our institution consistently. Before updating clinical guidelines for RSV bronchiolitis, the two tertiary hospitals adopted the national clinical guidelines for Bronchiolitis of 2014 (pre-protocol) [10]. In December of 2016, the following changes to the existing guide were made (post-protocol), following the evidence published at the time [14-23].

**Bronchodilators:** 1) previous recommendation: Short-acting beta2-adrenergic bronchodilators are not recommended, either on an outpatient basis or for treatment in hospital, in children under 2 years of age. 2) New recommendation: Monitored trial with short-acting beta 2-adrenergic bronchodilators in children with moderate or severe bronchiolitis (Wood´s Clinical Asthma Score) that does not improve with oxygen therapy, especially with positive asthma predictive index. 3) Saline hypertonic solution was not indicated. 4) Previous recommendation: The use of nebulized hypertonic saline solution(HSS) is recommended in the hospital treatment of children under 2 years of age. 5) New recommendation: The use of nebulized hypertonic saline solution(HSS) is not recommended for the treatment of children under 2 years of age.

**Chest X-rays and white blood cell (WBC) count:** 1) previous recommendation: chest X-ray and white (WBC) count are not recommended in the initial evaluation of children under 2 years of age. 2) New recommendation: chest X-ray and white (WBC) count are not recommended in the initial evaluation of children under 2 years of age and were only indicated if severe complications are suspected, in patients without favorable outcomes or in cases of diagnostic uncertainty.

**Statistical analysis:** continuous variables were presented as mean ± standard deviation (SD) or median (interquartile range [IQR]), whichever is appropriate. Categorical variables are shown as numbers (percentage). Differences between continuous variables were analyzed using the unpaired t-test or Wilcoxon's signed-rank test, whichever was appropriate. Associations between categorical variables and the outcome variables were analyzed using the chi-square test or Fisher's exact test, as needed. All statistical tests were two-tailed, and the significance level used was p < 0.05. The data were analyzed with Statistical Package Stata 15.0 (Stata Corporation, College Station, TX). A minimum sample size of 79 patients was estimated to estimate a minimum 20% reduction in bronchodilator use before and after, assuming a power of 80% and an alpha error of 5%.

**Ethical considerations:** the study protocol was reviewed and approved by the Institutional Review Board of the University of Antioquia (No 18/2015).

## Results

**General characteristics:** a total of 108 patients with RSV bronchiolitis were included in December of 2016 (pre-protocol group), and 92with RSV in December 2018 (post-protocol group). The demographic characteristics and clinical manifestations were similar in both groups (Table 1).

**Table 1:** characteristics of study population

Characteristics	2016 Pre-protocol	2018 Post-protocol	p
**Sociodemographic and comorbidities**			
Age< 6 months (%)	75(69.44)	64(69.57)	0.98
Male (%)	63(59.33)	54(58.70)	0.95
Prematurity(%)	18(16.67)	11(11.96)	0.34
Comorbidities (CHD or neurological)(%)	7(6.48)	3(3.26)	0.29
BPD (%)	3(2.78)	2(2.10)	0.10
Atopy (%)	1(0.93)	4(4.45)	0.12
Parental smoking (%)	9(8.33)	14(15.22)	0.12
**Signs in emergency department**			
Chest retractions(%)	62(57.41)	43(46.74)	0.13
Respiratory rate> 60(%)	4(7.55)	13(14.13)	0.23
Oxygen saturation<90% (%)	62(57.41)	59(64.13)	0.33
Temperature ≥38°C, %	26(24.07)	27(29.35)	0.40
**Laboratory parameters**			
Abnormal X-ray* (%)	30(28.04)	34(36.96)	0.179
Leucocytosis (> 15.000/mm^3^) (%)	13(12.04)	8(8.70)	0.44
Increased C-reactive protein (> 4 mg/lit.) (%)	67(62.04)	74(80.43)	0.004
**Outcome**			
length of hospital stay, median (IQR)	4[35]	4.4(3.51)	0.32
Pneumonia(%)	17(15.74)	27(29.35)	0.02
Bacteremiaor Sepsis (%)	10(9.26)	2(2.17)	0.03
Atelectasis (%)	2(1.85)	1(1.09)	0.65
Mechanical ventilacion (%)	11(10.19)	17(18.48)	0.09

CHD : Chronic heart disease; BPD: Bronchopulmonary dysplasia

**Outcomes:** the length of hospital stay was similar in both groups. The frequency of pneumonia was greater in the post-protocol group (15.74% vs 29.35%; p=0.02) although the frequency of bacteremia or sepsis was greater in the pre-protocol group (9.26% vs 2.17%; p=0.03).

**Diagnostic tests:** we did not find statistically significant differences in the performance of (WBC) count or chest X-rays, although the frequency of increased C-reactive protein (> 4 mg/lit.) was greater in the post-protocol group (80.43% vs 62.04%, p=0.004).

**Treatments:** there was a decrease in the use of salbutamol (67.3% pre-protocol vs 51.8 % post-protocol; P < .01). There were also significant reductions in the use of nebulized HSS (91.6% vs 82.6% P = .004). The prescription of antibiotics was similar in both periods (pre-protocol, 33.3%; post-protocol, 38.0%, p=0.48). Also, we did not find a statistically significant difference in the frequency of prescription of corticosteroids (pre-protocol, 12.9%; post-protocol, 14.1%, p=0.81) (Table 2).

**Table 2:** clinical outcomes and frequencies of use of medications and medical tests

Outcome	2016 Pre-protocol	2018 Post-protocol	p
**Salbutamol (%)**	72(67.39)	47(51.85)	0.03
**Hypertonic saline solution(%)**	99(91.67)	76(82.61)	0.04
**Corticosteroids (%)**	14(12.96)	13(14.13)	0,81
**Antibiotics (%)**	36(33.33)	35(38.04)	0,48
**X-ray (%)**	92(85.19)	86(93.48)	0,07
**WBC count (%)**	100(92.59)	88(95.65)	0,36
**C-reactive protein (%)**	79(73.15)	74(80.43)	0,22

## Discussion

The objective of this study was to determine the impact of the updating of clinical guidelines for RSV bronchiolitis on the use of diagnostic testing and medications in tertiary hospitals in Colombia. This update was effective, as it achieved decreases in the use of bronchodilators and nebulized HSS, without associated increases in length of stay or frequency of acute complications. We found a decrease in the percentage of patients that received a prescription for salbutamol of 15% and HSS of 9%. Demographic and clinical characteristics of the patients and the severity of RSV bronchiolitis were very similar in the 2 groups, allowing comparability between them.

The positive impact of the new protocol was consistent with the findings of other studies. Parikh *et al*. analyzed data of 41 pediatric hospitals in the US, to determine the impact of guidelines 2006 of APP. Multivariate regression analysis demonstrated differences in rates of change before and after guidelines, with significant improvement for bronchodilators (Preguideline: 64.6%, Post guideline 58%, p < 0.001) [16]. Despite documented variability between hospitals in the management of bronchiolitis in the US [24], these tendencies were higher in pediatric-specific emergency departments than in general emergency departments [25]. Johnson *et al*. analyzed the impact of 2006 APP guidelines on bronchiolitis on medication usage and diagnostic tests in 678 patients of general hospitals in the US. The use of bronchodilators did not change significantly over the study period (53.6% vs 54.2%; p=0.91) [26]. Garcia *et al*., in a retrospective study of 113 patients with bronchiolitis, analyze the differences in the use of non-recommended resources in the management of bronchiolitis, before (December 2014) and after (December 2016) the establishment of a new protocol. There was a significant decrease in the use of salbutamol, both in the Emergency Department (33.6% vs 19.5%, P < 0.01) and at discharge (46.7% vs 15.2%, P < 0.001); and nebulized hypertonic saline solution(5.3% vs 0.8%, P = 0.04) [26].

Breakell *et al*. in a General Hospital in England, evaluated in 101 patients the effect of implementing the NICE bronchiolitis guideline, accompanied by an educational program, on the frequency of use of medical tests, antibiotics, and nebulized treatment in 2014-2015. As a result, antibiotics reduced by more than threefold (from 22 to 6% of patients; absolute reduction 16%) and inhaled/nebulized treatment up to twofold (from 30 to 16%; absolute reduction 14%). Overall NICE guideline compliance rose from 28 to 63% [27]. Accompanying educational interventions with guideline changes are effective in reducing inappropriate antibiotic and medical test use. Tyler *et al*. revealed a significant reduction, after educational interventions, in the ordering of chest radiographs (from 22.7% to 13.6%; P ≤ 0.001), respiratory viral testing (from 12.5% to 9.8%; P = 0.001), and bronchodilators (from 17.5% to 10.3%; P = 0.001) without changes in balancing measures (eg, hospital read mission within 7 days (1.7% (pre-analysis) and 1.0% (post-analysis); P = 0.21)) [28]. Despite that few physicians are compliant with the bronchiolitis guidelines[29], updating of guidelines and educational interventions improves the patterns of prescription of drugs and diagnostic tests, both in developed and developing countries, as we have shown in our study.

In our study, the impact of the new protocol on the prescription of diagnostic tests was not significant. While two studies in the US [16,30] and others in the UK [27] showed reductions in the prescription of x-rays, another study in Spain was not able to demonstrate such reductions. It could even be expected that in our study the frequency of x-rays would be higher post-protocol due to the greatest number of pneumonia cases post-protocol; however, this did not happen, perhaps in part due to the restriction of the new protocol. As occurred in other studies, the new protocol did not achieve a reduction in the prescription of antibiotics [16,26,27,30]. As had occurred before with bronchodilators, the frequencies of antibiotics were similar than reported in US [16,30], and higher than reported in Spain and the UK [26,27].This is explained by the great influence of the AAP guidelines on the recommendations of local guidelines in Latin America. The frequency of antibiotic prescriptions does not correlate with the proportion of cases of pneumonia. For example, pre protocol, 33.3% of the patients used antibiotics, but only 25% of the patients had some diagnosis of infectious disease. Post-protocol, 38% used antibiotics, and 31% had some diagnosis of infectious disease. This is evidence of misuse of antibiotics in a viral disease such as RSV bronchiolitis; already documented also in developed countries [31,32].

Our study has limitations. First, since this study was based on a medical records review, we cannot include other variables such as environmental pollution and genetic factors, and residual confounding cannot be excluded. Second, the study was conducted in a tertiary referral hospital, and therefore, the patients included represented the high spectrum of severity, limiting the generalization of results to other contexts. However, the similarity of our population in terms of clinical characteristics, risk factors, and seasonality of bronchiolitis in our country with previous reports suggests strength and consistency in our results [33,34]. Third, in our study, we used an immunofluorescence assay for the diagnosis of RSV infections that, despite being widely available, was easy to perform. But we did not determine the RSV genomic load, and also we did not test for viruses. This can generate some differential misclassification bias, which could have overestimated the true association between RSV isolation and the outcome variable; however, the previous evidence in other populations had confirmed this association being plausibility of our results.

## Conclusion

Our results demonstrate that the updating of clinical guidelines for RSV bronchiolitis was effective, as it achieved decreases in the use of bronchodilators and nebulized hypertonic saline solution. It is necessary to continue developing new strategies targeted to increase adherence to guidelines and evaluate the impact on the use of resources.

### What is known about this topic

- Clinical practice guidelines are useful tools to reduce variability in clinical care;
- Few studies have evaluated the impact of recent updates to clinical practice guidelines on clinical outcomes.

### What this study adds

- Our results demonstrate that the updating of clinical guidelines for RSV bronchiolitis was associated with decreases in the use of bronchodilators and nebulized hypertonic saline solution; it is necessary to continue developing new strategies targeted to increase adherence to guidelines and evaluate the impact on the use of resources;
- The results of this study could be used as an input for the construction of compliance indicators in future versions of the clinical guidelines for RSV bronchiolitis.
